# Postural orthostatic tachycardia syndrome and other common autonomic disorders are not functional neurologic disorders

**DOI:** 10.3389/fneur.2024.1490744

**Published:** 2024-11-20

**Authors:** Svetlana Blitshteyn, Glenn J. Treisman, Ilene S. Ruhoy, David S. Saperstein, Jill R. Schofield, Brent P. Goodman, Todd E. Davenport, Alexis C. Cutchins, Blair P. Grubb

**Affiliations:** ^1^Department of Neurology, University of Buffalo Jacobs School of Medicine and Biomedical Sciences, Buffalo, NY, United States; ^2^Dysautonomia Clinic, Williamsville, NY, United States; ^3^Department of Psychiatry, Johns Hopkins School of Medicine, Baltimore, MD, United States; ^4^Department of Neurology, Mount Sinai South Nassau, Oceanside, NY, United States; ^5^Center for Complex Neurology, University of Arizona College of Medicine, Phoenix, AZ, United States; ^6^Department of Medicine, University of Colorado, Aurora, CO, United States; ^7^Metrodora Institute, West Valley City, UT, United States; ^8^Department of Physical Therapy, University of the Pacific, Stockton, CA, United States; ^9^Division of Cardiology, Emory University School of Medicine, Atlanta, GA, United States; ^10^Division of Cardiovascular Medicine, University of Toledo, Toledo, OH, United States

**Keywords:** postural orthostatic tachycardia syndrome (POTS), neurocardiogenic syncope, orthostatic hypotension (OH), inappropriate sinus tachycardia (IST), functional neurologic disorder, autonomic disorders, dysautonomia, diagnostic criteria

## Introduction

In the past 4 years of COVID-19 and Long COVID, a renewed interest in postural orthostatic tachycardia syndrome (POTS) and other autonomic disorders brought to light a common misconception that these disorders are based in or are associated with functional neurologic disorder (FND). Recently, one narrative review attempted to link autonomic disorders and autonomic nervous system dysfunction with symptoms of FND (1). Others have similarly suggested that Long COVID may be based in functional or somatic etiology (2–5). As medical professionals with expertise in autonomic disorders, we would like to emphasize the distinction between autonomic disorders, autonomic symptoms and FND in order to ensure that appropriate diagnostic and therapeutic pathways are implemented by clinicians.

## POTS and other autonomic disorders have diagnostic criteria distinct from FND

First, it's important to highlight that POTS and other common autonomic disorders are not functional neurologic disorders and are not based in functional etiology. Objective diagnostic criteria for common autonomic disorders have been established: orthostatic intolerance with postural tachycardia, persistent sinus tachycardia, orthostatic hypotension or a fall in blood pressure, heart rate and cerebral perfusion must be present in common autonomic disorders (6–8), while none of these features are present in the diagnostic criteria of FND (Table 1) (9). A 10-min stand test or a tilt table test is required to diagnose common autonomic disorders (6–8), but FND diagnosis lacks vital signs diagnostic testing and is based primarily on clinical assessment (9). Conversely, alterations in voluntary motor or sensory function—a defining diagnostic criterion for FND—are not present in the diagnostic criteria of POTS and other common autonomic disorders (9) (Table 1).

**Table 1:** Diagnostic criteria for common autonomic disorders and FND.

**Disorder**	**Diagnostic criteria**
**Autonomic**	
POTS [6–8]	1. HR increase ≥30 bpm within 10 min for adults (≥40 bpm for adolescents 12–19 years of age) of standing or TTT 2. Absence of OH 3. Symptoms of orthostatic intolerance for ≥3 months 4. Exclusion of other causes of postural tachycardia, such as dehydration, medication side effect and other medical conditions
NCS [6, 7]	1. Transient loss of consciousness typically preceded by prodromal symptoms and signs, such as pallor, diaphoresis, nausea, abdominal discomfort, yawning, sighing, and hyperventilation. that may occur up to 60 s prior to loss of consciousness. 2. A sudden fall in blood pressure, heart rate and cerebral hypoperfusion on standing or TTT
OH [7]	Sustained drop in blood pressure ≥20/10 mmHG within 3 min of standing or TTT
IST [6]	1. Average sinus HR exceeding 90 bpm over 24 h or HR while awake and at rest ≥100 bpm 2. Palpitations and other distressing symptoms associated with sinus tachycardia
**Functional neurologic [9]**	• One or more symptoms of altered voluntary motor or sensory function • Clinical findings provide evidence of incompatibility between the symptom and recognized neurological or medical conditions • The symptom or deficit is not better explained by another medical or mental disorder • The symptom or deficit causes clinically significant distress or impairment in social, occupational or other important areas of functioning, or warrants medical evaluation

POTS, postural orthostatic tachycardia syndrome; NCS, neurocardiogenic syncope; OH, orthostatic hypotension; HR, heart rate; bpm, beats per minute; TTT, tilt table test; IST, inappropriate sinus tachycardia.

## Common comorbidities of POTS

Second, data on POTS from three different studies have not identified FND to be a common comorbidity of POTS among a combined cohort of 5,214 patients (10–12). The most common comorbidities of POTS are well-established and include migraine (at least 40%), gastrointestinal disorders (at least 30%), small fiber neuropathy (at least 50%), Ehlers-Danlos syndrome and hypermobility spectrum disorders (HSD) (at least 30%), autoimmune disorders (at least 20%) and mast cell activation syndrome (at least 20%) (10–12). Furthermore, in a study of 526 participants with FND, only three patients had POTS before FND diagnosis and seven patients were diagnosed with POTS after FND diagnosis, making POTS one of the rarest FND comorbidities, occurring at 1.9% (13). By contrast, among study participants with FND, migraine occurred with a prevalence of 11.5% (13). It's important to mention that these studies did not specifically assess the prevalence of FND in patients with POTS or prevalence of POTS in patients with FND (10–13). Further studies are needed to determine whether FND and POTS are comorbid conditions and if they are, what percentage of patients satisfy the diagnostic criteria for both disorders. Additionally, studies on prevalence of FND in patients with POTS vs. the general population would be informative to determine whether patients with POTS are at a higher risk of developing FND compared to age- and sex-matched healthy controls.

## Autonomic disorders vs. autonomic symptoms

Third, while central autonomic networks may be activated in certain psychiatric diseases, including panic disorder, PTSD and generalized anxiety disorder, autonomic disorders should not be conflated with autonomic symptoms that might accompany these psychiatric disorders. Autonomic symptoms when present in patients with FND may not specifically signify a comorbid autonomic disorder. As mentioned above, the diagnostic criteria for common autonomic disorders are based on the objective parameters of vital signs in conjunction with orthostatic intolerance (6–8), not just the autonomic symptoms reported by the patient. In contrast, there are no heart rate and blood pressure parameters and no requirement for orthostatic intolerance in the diagnostic criteria of FND (Table 1) (9).

## Discussion

The autonomic nervous system is important in health and disease, including in the stress response to physiologic and psychological stressors. While it may be tempting to combine many chronic diseases manifesting with chronic fatigue and chronic pain under the umbrella of “functional” etiology, refraining from making these sweeping and unfounded generalizations that blur the lines between diagnoses and etiologies is essential. Importantly, we emphasize the importance of utilizing diagnostic criteria for FND, POTS and other autonomic disorders to avoid confusion and misdiagnosis among clinicians, researchers and patients (6–9) (Table 1). In cases where a patient may have both POTS and FND based on meeting the diagnostic criteria for both disorders, we recommend treating POTS first with appropriate pharmacologic and non-pharmacologic therapies before referring the patient for FND-targeted psychotherapy and physical therapy.

In our experience, many patients with autonomic disorders, including those with post-COVID dysautonomia as part of Long COVID, post-treatment Lyme disease syndrome and other infection-associated chronic illnesses, are frequently misdiagnosed with FND (14). Once diagnosed with FND, patients often have difficulty obtaining further diagnostic and therapeutic care because of attribution of any further complaints to the psychiatric diagnosis.

Importantly, some authors suggest that pathophysiology of FND stems from emotion processing, agency, attention, interoception, and predictive processing/inference, with underlying neural circuits to include salience, multimodal integration, and attention networks (15). Autonomic dysfunction is not thought to be mechanistically related to FND and is not a typical clinical feature in patients with FND. Conversely, these cortical functions are not the accepted pathophysiologic mechanisms of POTS, orthostatic hypotension, inappropriate sinus tachycardia or neurocardiogenic syncope. Importantly, pathogenic mechanisms of common autonomic disorders include hypovolemia, small fiber neuropathy, cerebral hypoperfusion, autoimmunity and mast cell hyperactivity (16–18) whereas the etiology of FND is thought to be due to non-structural causes.

Finally, treatment of common autonomic disorders includes salt and fluid supplementation, specialized recumbent and supine exercise programs and pharmacologic interventions with beta blockers, vasoconstrictors, aldosterone analog, parasympathetic nervous system enhancers and sympatholytics, as well as other agents targeting the underlying mechanisms of autonomic disorders (16–18). In contrast, treatment of FND focuses on psychotherapy and physical therapy as the sole therapeutic modalities. While exercise is often beneficial in many patients with autonomic, cardiovascular and neurologic disorders, it is typically prescribed in conjunction with, not exclusive of, targeted symptomatic or disease-modifying therapies.

Exercise intolerance is one of the key features of autonomic dysfunction rendering many patients unable to participate in exercise programs conducted in an upright position. Furthermore, the presence of post-exertional malaise as a key feature of myalgic encephalomyelitis/chronic fatigue syndrome (ME/CFS) or joint hypermobility as a feature of HSD, both of which are comorbid with POTS, can make physical therapy unhelpful, and in some cases, detrimental when administered in the absence of ME/CFS or HSD diagnoses and a corresponding expertise of an experienced healthcare professional. In contrast, patients with FND do not typically have restrictions or specifications for exercise required for patients with autonomic disorders, ME/CFS and HSD.

## Conclusion

In summary, diagnostic criteria for POTS and other common autonomic disorders have been established and are vastly different from the diagnostic criteria of FND (Table 1) (6–9). Pathophysiology of and therapies for POTS and FND are also vastly different. Taken together, we emphasize that POTS and other common autonomic disorders are not FND and are not “functional” in etiology. Additionally, we believe that uniformly recommending FND-targeted exercise for patients who are misdiagnosed with FND or diagnosed with FND in conjunction with an autonomic disorder, is inappropriate and not in line with the current diagnostic criteria and scientific evidence. Further studies on pathophysiology, prevalence and therapeutic options for patients with POTS and patients with FND are needed as both conditions are complex and highly disabling.
